# Surgery and Otology

**Published:** 1875-11

**Authors:** 


### IV. Surgery and Otology.

1. *Rupture of External Iliac Artery.* John Ashhurst, Jr., M.D. (*Phila. Med. Times*, Aug. 21, 1875.)

An engineer, 39 years of age, applied for admission to the Episcopal Hospital, on June 15, saying that one week before he had, in trying to board a railway train, slipped and fallen backward, striking a rail and receiving a severe blow in the region of the lumbar spine. Slight inconvenience from the injury was experienced at the time, but the next morning the patient felt stiff, and had a good deal of pain in the back. When admitted to the hospital he walked without difficulty and was considered to be suffering merely from a contusion or sprain of the lumbar muscles. On the following day, the patient was in much the same condition and gave no evidence of having received any serious injury. On June 17, I found the patient in a cold sweat, and then learned that a similar "sinking spell" had occurred on the preceding day, but had passed off in a short time. The patient complained of pain in the left side as well as in the back, but there was no tenderness on palpation. The left common femoral artery was observed to be pulsating with great force. On June 18, the patient was almost in a state of collapse, bathed in a profuse, cold sweat, with rapid respiration and with a pulse running at the rate of 120 or more beats in the minute; he had more pain, still referred to the back and left flank, and vomited a little after taking food. There was no tympanitis and no general abdominal tenderness, and the tongue was moist and clean. Pulsation in the common femoral artery was now rather feeble, but a marked pulsation in the epigastric region was perceptible both to sight and to touch; auscultation, moreover, revealed a bruit, not however, of a very decided character, and not propagated for any great distance along the course of the aorta. There was no bruit in the iliac region. Careful examination failed to discover a tumor in any part of the abdomen, though there was a slight fullness in the left flank. On June 19, there was a good deal of tympanitis, but no general abdominal tenderness; vomiting had ceased; three copious fecal evacuations followed a simple enema. That which the day before was only a fullness in the left flank, now appeared to form an elongated tumor; the pain and tenderness on the left side were still increased. Patient died, and a post-mortem examination was made the next day. On laying open the abdomen, recent peritoneal adhesions were found upon the left side, and beneath the peritoneum was found an enormous clot, filling the left vertebral gutter from the pelvis to the diaphragm, and above, turning inward and compressing the aorta. The clot having been removed, the source of bleeding was found in a rupture of the left external iliac artery, about half an inch below the bifurcation of the common trunk; both ends of the vessel were retracted, though held together by a portion of the external coat of the artery, and the distal end was partially occupied by a well formed coagulum. An incomplete rupture of the external iliac artery no doubt occurred at the time of the patient's injury; the bleeding was temporarily arrested, but recurred on the eighth or ninth day; the blood found its way upwards, dissecting up the peritoneum (giving rise to the steadily increasing pain, and ultimately leading to peritonitis) and finally impinging upon the aorta, thus by pressure producing the pulsation and bruit.

2. *Fusiform Aneurism of the Anterior and Posterior Tibial Arteries.* De Forrest Willard. (*Phila. Med. Times*, Sept. 25, 1875.)

The following case is published as another evidence of the superiority of compression over ligature. A shoemaker, aged 66, began in June to complain of pains in both legs below the knees. They gradually increased and became almost unendurable. On July 10, pulsation of the calf of the left limb was noticed for the first time, and on the 15th the following notes were taken: "The hand placed upon any portion of the leg from the insertion of the soleus upon the tendo Achillis, to the knees, perceives most decidedly the characteristic thrill, while the fibula is forcibly thrust against the finger. There is no well defined and limited tumor, but a general enlargement of the entire leg. The pulsation is strongest over the lines of the two main arteries, and the ear along their course, plainly detects a loud bruit. There is no undue pulsation of the popliteal, nor do the thrill and bruit extend to that artery. The dilatation evidently commences just at the point where the bifurcation occurs. Both arteries are largely dilated, but taper towards the ankle, where the normal calibre is resumed. Pressure upon either the femoral or popliteal artery entirely arrests all pulsation, thrill and bruit, and also relieves the pain." All arteries are atheromatous ; and the patient is suffering from albuminuria of long standing. Ligation would have been, at best, dangerous ; digital compression of the femoral artery was therefore advised, and commenced upon the 17th of July ; and uninterruptedly continued until the end of fifty-five hours. The thrill ceased at the end of twenty hours, and the pulsation stopped at thirty-six hours. Sixteen persons were alternately engaged in performing the compression.

3. *Treatment of Varix by Local Application of Perchloride of Iron.* DR. LINON. (*L'Independente ; N. Y. Med. Journal*, Oct., 1875.)

Dr. Linon (Verviers) has for three years treated varix with great success by the local application of perchloride of iron. He used a solution of the strength of six grammes in two hundred of water (about 1¼ drachms in 6⅝ ounces). Compresses of flannel are to be wrung out in this fluid, and applied to the parts with a moderately tight bandage. These applications should be renewed for seven or eight days consecutively, after which the bandage should be continued without further wetting with the solution. After a time, the solution is again used, and the bandage is then applied and left till the varix has entirely disappeared, which generally occurs in one or two weeks, according to the extent of the swelling.

4. *Treatment of Ununited Fractures.* SPENCE. (*British Med. Journal*, Aug. 14.)

Discussing the various operations for the cure of ununited fractures, at a meeting of the British Med. Assoc., Prof. Spence said: "There is a method of treating ununited fractures, especially at an early period, which I do not think has had sufficient notice taken of it, nor been sufficiently or fairly tried; I mean the plan proposed and practiced by the late Professor Miller. The method consists in entering subcutaneously a long, narrow but strong knife; passing it on to and between the ends of the ununited bone, dividing freely the fibrous union, scraping the ends of the bone, and slightly separating the periosteum; then the limb is carefully and firmly bandaged, and placed in appropriate splints. I know that this plan has never had much favor; perhaps it seemed too simple to effect the purpose, and it was derided and declared to be inefficacious by some who professed to have tried it. But I also know that I have frequently used it, and have generally found it successful when the knife has been effectively used, and the after-treatment carried out carefully. I have, indeed, so strong an opinion of the efficacy of this method in comparatively recent cases, that I do not think it warrantable to proceed to severer measures until this simple one has been fairly tried, and I purposely mention it here to press it on the attention of the profession."

5. *A New Method of Preparing Plaster of Paris Bandages.* ED. J. FORSTER. (*Boston Med. and Surgical Journal*, Oct. 7, 1875.)

To simplify the preparation of such bandages, Forster had a pan made, into which could be inserted the common bandage roller. The pan is partly filled with the dry plaster; and, when the bandage is rolled, it will be covered on both sides with the plaster, and the latter is evenly distributed and forced into the meshes of the cloth by the rod of the roller under which the bandage passes. The method is distinguished by the ease and neatness with which a bandage can be prepared.

[From our own experience we can highly recommend this method, for we have seen it employed in the University Hospital, at Heidelberg, in 1862.—Ed.]

6. *Sulphuric Acid in the Treatment of Boils.* Dr. M. Marsh. (*Practitioner*, Aug., 1875.)

Dr. Marsh has used sulphuric acid, which he regards as almost a specific for boils, with constant success for twenty-five years. "As soon as the patient applies for relief, I put an adult on elixir vitriol, 20 drops, three times a day, in a glass of sweetened water, one hour before meals, previously smearing the teeth well with fresh butter, in order to protect them. This is much better than sucking through a quill, and is a perfect protection, if the teeth are subsequently washed with a solution of bicarb. sod., a heaped teaspoonful to a glass of water. In the use of sulphuric acid the boil will soon melt away and disappear, no more to reappear. The acid should be kept up in ten-drop doses for at least two weeks after the boils have disappeared. To afford relief from pain and soreness, I apply a piece of common adhesive plaster, cut round, sufficiently large to cover the tumor, clipping the edges so that it will set smooth."

7. *Foreign Body in the Larynx.* L. Schroetter. (*Ohio Med. and Surg. Reporter*, Sept., 1875.)

The following case, related by Dr. L. Schroetter, in his lately published "Report of the Clinic for Laryngoscopy at the University of Vienna," is most extraordinary. A very intelligent man noticed at breakfast that his false teeth (four upper incisors attached to a gold plate) were missing. When he began to search for them, he felt for the first time some obstruction in the throat and some difficulty in breathing, and then concluded that he must have swallowed the teeth during the night. He had taken on the previous evening only a light meal, without much alcohol. Dr. S. saw the patient the same day. Laryngoscopic manipulation was difficult, and a superficial examination showed nothing abnormal. The foreign body was therefore supposed to be in the œsophagus, but in passing a probang nothing was brought up. A very careful examination of the larynx was made, and after several trials a foreign body was seen under the vocal cords, and soon the plate and two teeth were made out with certainty. S. urged immediate laryngotomy, but Dr. Czerny thought that as Schroetter saw the foreign body so clearly, he might first attempt its removal through the mouth by the aid of the laryngoscope. S. consented, and appointed 5 P. M. for the operation. However, when he returned at that hour, he learned that during his absence the dyspnœa had increased so rapidly that Dr. Czerny had been hastily summoned about 2 P. M., and had been obliged to perform laryngotomy at once, the patient's breathing being suspended during the operation. On dividing the crico-thyroid membrane, the point of the bistoury struck the artificial teeth. An attempt was made to remove the foreign body through the wound, but not succeeding in this, Dr. Czerny forced it up through the glottis and removed it through the mouth. The patient wore a tube for thirteen days, and made a good recovery.

8. *Tinnitus Aurium.* SAMUEL THEOBALD, M.D. (*Trans. Med. and Chirurg. Faculty of Maryland*, April, 1875.)

Imaginary sounds are a symptom common to almost all, and peculiar to none, of the diseases of the ear; and besides, a long list of pathological conditions of a more general character, and of constitutional disturbances, might be given, in which the tinnitus aurium often plays a prominent rôle. The theory at present generally accepted to account for the tinnitus aurium is, that it caused by abnormal pressure upon the peripheral expansion of the auditory nerve in the labyrinth, such as would result from the stapes being pressed in upon the labyrinthine fluid. Where the presence of this symptom cannot be attributed to this cause, as is often the case, it is customary to assume the existence of a morbidly increased irritability of the auditory nerve, an irritation of the auditory nerve, whether of its trunk or its terminal expansion in the labyrinth. Dr. Theobald now thinks, in the first place, that the tinnitus is invariably due to an excitation of the *terminal* elements (not the trunk) of the auditory nerve, and, after a careful consideration of the various physical, anatomical and pathological facts which seemed to have any bearing on the subject, he has been led to the conclusion, "that, in almost all cases, tinnitus aurium, whether associated with aural affections, cerebral diseases or constitutional disorders, is to be attributed to the existence of *vibrations excited in the walls of the blood-vessels of the labyrinth, by the friction attending the circulation of the blood*, which are capable of imparting to the labyrinthine fluid, and thence to the terminal fibres of the auditory nerve, impulses similar in character to those which are produced by the vibrations of the stapes, and hence, like them, capable of giving rise to the sensations of sound.

The vibrations of these vessels can produce a sensible impression upon the auditory nerve in two ways. First, the intensity of the vibrations may be increased. Second, the vibrations remaining unaltered, their effect upon the nerve may be magnified by reflection and concentration or by resonance. The first condition will obtain whenever the natural and easy flow of the blood is perturbed, as for instance in hyperæmia or anæmia of the labyrinthine vessels, by partial compression of the vessels by inflammatory or other causes; especially by an incursion of the stapes by which the tension of the labyrinthine fluid is increased. By the second mode—reflection or resonance—the tinnitus is produced, which accompanies the affections of the middle and external ear. The conducting apparatus of the ear is defective; therefore sonorous undulations from without, fail to reach the auditory nerve, and, on the other hand, sonorous vibrations arising in the ear are not allowed to escape; they are reflected, concentrated, and their sound magnified. The experiment with a tuning-fork is referred to for demonstration. If it is placed upon the middle of the forehead and one ear closed with the finger, the sound of the fork will be heard only by this ear and much louder than before.

